# Circulating sepsis-related metabolite sphinganine could protect against intestinal damage during sepsis

**DOI:** 10.3389/fimmu.2023.1151728

**Published:** 2023-05-24

**Authors:** Zetian Wang, Yue Qi, Fei Wang, Baiyin Zhang, Tang Jianguo

**Affiliations:** Department of Trauma-Emergency & Critical Care Medicine, Shanghai Fifth People’s Hospital, Fudan University, Shanghai, China

**Keywords:** sepsis, serum metabolomics, machine learning, sphinganine, intestinal barrier function

## Abstract

**Introduction:**

Sepsis is intricately linked to intestinal damage and barrier dysfunction. At present times, there is a growing interest in a metabolite-based therapy for multiple diseases.

**Methods:**

Serum samples from septic patients and healthy individuals were collected and their metabonomics profiling assessed using Ultra-Performance Liquid Chromatography-Time of Flight Mass Spectrometry (UPLC-TOFMS). The eXtreme Gradient Boosting algorithms (XGBOOST) method was used to screen essential metabolites associated with sepsis, and five machine learning models, including Logistic Regression, XGBoost, GaussianNB(GNB), upport vector machines(SVM) and RandomForest were constructed to distinguish sepsis including a training set (75%) and validation set(25%). The area under the receiver-operating characteristic curve (AUROC) and Brier scores were used to compare the prediction performances of different models. Pearson analysis was used to analysis the relationship between the metabolites and the severity of sepsis. Both cellular and animal models were used to HYPERLINK "javascript:;" assess the function of the metabolites.

**Results:**

The occurrence of sepsis involve metabolite dysregulation. The metabolites mannose-6-phosphate and sphinganine as the optimal sepsis-related variables screened by XGBOOST algorithm. The XGBoost model (AUROC=0.956) has the most stable performance to establish diagnostic model among the five machine learning methods. The SHapley Additive exPlanations (SHAP) package was used to interpret the XGBOOST model. Pearson analysis reinforced the expression of Sphinganine, Mannose 6-phosphate were positively associated with the APACHE-II, PCT, WBC, CRP, and IL-6. We also demonstrated that sphinganine strongly diminished the LDH content in LPS-treated Caco-2 cells. In addition, using both in vitro and in vivo examination, we revealed that sphinganine strongly protects against sepsis-induced intestinal barrier injury.

**Discussion:**

These findings highlighted the potential diagnostic value of the ML, and also provided new insight into enhanced therapy and/or preventative measures against sepsis.

## 1 Introduction

Sepsis is a widespread acute disease that causes severe multi-organ dysfunction syndrome (MODS) and circulatory failure (1, 2). The injuried intestinal induced by sepsis, which further aggravates septic development, eventually results in severe infection, and even death (3, 4). Currently, the primary protective measures against sepsis-induced intestinal injury are modulation of the intestinal flora disorder, along with the early initiation of enteral nutrition. However, the associated therapeutic effect is relatively unsatisfactory (5). Given the previous evidences, protecting the intestine from sepsis-induced damage is crucial to the prevention and therapy of sepsis itself.

Recent investigations highlighted a strong role of patient metabolism in modulating cellular function, and this is intricately linked to the development and pathogenesis of multiple diseases (6). Several circulating metabolites have been identified as possible diagnostic and prognostic indicators of different diseases (7, 8). A European research team, for example, reported that multiple serum metabolites, phosphatidylcholines, sphingomyelins, triglycerides, amino acids, and cholesteryl esters, are heavily altered in Hepatocellular carcinoma (HCC), and that several of these metabolites exhibit enhanced diagnostic sensitivity and specificity, compared to alpha-fetoprotein(AFP) (9). Notably, the protective function of several metabolites have been identified. One such example isγ-aminobutyric acid(GABA), which suppresses Reactive Oxygen Species(ROS) generation and monocyte adhesion to protect cells and tissues against cardiovascular disease (10).

The diagnostic indicators of sepsis mainly include body temperature, heart rate, respiratory rate, white blood cell count, serum C-reactive protein (CRP), and procalcitonin (PCT) and other biochemical indicators. However, these indicators often lack specificity in many cases and cannot determine whether a patient has sepsis. To improve the diagnostic accuracy of sepsis, researchers are currently exploring new diagnostic indicators, such as cell surface receptors, cytokines, metabolites, etc. Over the past decade, machine learning (ML) has gained remarkable interest in biomedical research for its potential to provide computer-aided diagnoses of various diseases (11). Machine learning techniques can enhance the predictive power of disease prediction models, notably the blood pressure neural network, which can be used to exploit genomic information for the discovery of molecular markers, as well as to aid in the identification of distinctive methylation sites in stomach cancer (12). Herein, we employed metabolomics to compare between the serum samples of septic patients and healthy individuals. Machine learning was used to screen differential metabolites and construct a diagnostic model to predict the diagnostic value of metabolites in sepsis. Lastly, we also explored the metabolite-mediated protection of intestinal barrier using both *in vitro* and *in vivo* experimentations.

## 2 Materials and methods

### 2.1 Clinical sample collection

Human samples were retrieved from healthy individuals (n =13), who were the volunteer population from health check-up center and septic patients (n = 13), who sought treatment at the Shanghai Fifth People’s Hospital. This research received ethical approval from the aforementioned institution (Reference No. 2019-118), and informed consent from the legal guardians of study subjects. The following septic paitents were included in analyses: those with (i) sepsis diagnosis, based on the Third International Consensus Definitions for Sepsis and Septic Shock (13); (ii)between the age of 18 and 80; and (iii) hospitalized in our department within 12 h of sepsis onset. Among septic patients excluded from analyses were those infected with the human immunodeficiency virus, and complicated with hematologic malignancies, or those who underwent immunosuppressive therapy within 1 month of the start of this investigation. Pregnant and lactating females were also eliminated from the study analyses. The following healthy individuals were included in our analyses: (a) age and gender matched with septic patients; (b) with no abnormality in biochemical indexes, which was confirmed in the health examination. Among the healthy individuals who were eliminated from this study were: those with (a) prior sepsis or other severe infections; (b) prior hematological malignancies or other solid tumors; and (c) complicated with inflammatory disease.

Serum samples (n=13) were collected within 12h of admission, and healthy individual samples (n=13) were taken following admission. All samples underwent a 10 min centrifugation at 1,500 r/min, prior to storage at −80°C.

### 2.2 Metabolomics analysis of serum

Diluted serum samples in 1-l aliquots were inserted into a Waters Ultra-Performance Liquid Chromatography-Time of Flight Mass Spectrometry(UPLC-TOFMS) machine (Milford, MA). Chemical components underwent separation at 35°C *via* an Acquity UPLC BEH C18 column (Waters). During a 10-minute run, the adjusted mobile-phase flow rate was 0.5 ml/min, and aqueous acetonitrile gradient contained 0.1% formic acid (0% acetonitrile for 0.5 min, 20% acetonitrile by 5 min, 95% acetonitrile by 9 min, followed by equilibration at 100% water for 1 min prior to subsequent administration). The Waters QTOF Premier mass spectrometer was adjusted to positive electrospray ionization. The capillary and cone voltages were maintained at 3 kV and 20 V, respectively. The source and desolvation temperatures were at 120°C and 350°C, respectively. Nitrogen was employed as the cone (50 l/h) and desolvation gas (600 l/h), whereas, argon was used as the collision gas. The flight mass spectrometry duration was calibrated using sodium formate solution (range m/z 100-1000), and observed in real time using intermittent administration of the lock mass sulfadimethoxine ([M + H]+ = 311.0814 m/z). Mass chromatograms and mass spectrum information were retrieved and assessed in the centroid format with the MassLynx program (Waters).

### 2.3 ML analysis

We used sequential linear regression models to establish correlations among the variables present in the dataset. Then extreme gradient boosting (XGBoost)was employed for relevant metastatic agent identification using python 3.7. The data set was randomly split into two data sets: a training (75%) data set, which was used to develop the models, and an internal validation (25%) data set, which was used to validate the constructed models. We utilized the following five representative ML classifier algorithms for model construction in the training data set: Logistic Regression, extreme gradient boost (XGBoost), GaussianNB(GNB), upport vector machines(SVM) and RandomForest. To ensure maximum use of data, we did use a crossvalidation method. The accuracy, precision (also called positive predictive value) and F1-score (F1) were calculated for each ML model to be evaluated and compared in the validation cohort. Through comprehensive evaluation of multiple evaluation indicators, the best performing model among the five ML models after using 5 cross- validations, was defined as the optimal model and selected for further prediction analysis. Finally, we performed calibration curve to evaluate the consistency of the optimal model.To build trust with healthcare professionals and make the decision-making process of machine learning transparent, it is important to understand how the model works. One way we did this was by using the SHAPELY Additive explanations (SHAP)values method to improve the interpretability of the best-performing model. SHAP values help us understand how each feature contributes to the model’s output and how they affect the final prediction.

### 2.4 Mouse models

We acquired 6-8 week-old C57BL/6 mice, weighing between 20-23 g, from the Animal Center of East China Normal University (Shanghai, China), and housed them in plastic boxes with ad libitum standard rodent food and water. The room temperature was adjusted between 20-22°C, with a 12-hour light/dark cycle. All animal protocols received ethical approval from the East China Normal University (Shanghai, China). Following 1-week of adaptive feeding, mice were arbitrarily separated into four groups as follows, mice were randomly divided into 4 groups, 20 mice per group [Control, Sepsis, Sepsis+sphinganine(10mg/kg, 15mg/kg and 20mg/kg, respectively), sphinganine 15mg/kg]. Sphinganine was dissolved in vehicle (10% dmso and 90% saline [1:9]), was administered intraperitoneally at 6 h and 12 h after surgery and puncture. At 24 h post-surgery, mice were euthanized and samples of fresh stool, blood and main organs were isolated.were collected immediately.

### 2.5 Cell culture and treatment

Human colorectal adenocarcinoma (Caco-2) cells were maintained in Eagle’s Minimum Essential Medium with 10% heat-inactivated fetal bovine serum (FBS, Gemini Bioproducts) and 1% non-essential amino acids from the American Type Culture Collection (Invitrogen, Manassas, VA, USA). Caco-2 cells (1 × 10^6^ cells/well) were grown in 6-well plates, prior to treatment with Salmonella enterica serotype Typhimurium lipopolysaccharide (LPS) (Sigma). With increasing dosage experimentation, we established the optimal LPS dosage to be 1 mg/mL for a duration of 48 h. Hence, our cell cultures underwent LPS stimulation, in presence or absence of 10μM mannose-6-phosphate (Selleck, USA), and 5μM, 10μM, and 20μM sphinganine (Selleck, USA), respectively.

### 2.6 Intestinal histomorphological analysis

To conduct histological analysis, intestinal tissues underwent a 24-hour fixation in 10% neutral-formalin in PBS, followed by paraffin-embedding, then slicing into 4m thick sections, and staining with hematoxylin and eosin (H&E). Finally, IA pathologist, unaware of the specifics of this investigation, employed a light microscope (Olympus CX30, Japan) to assess the intestinal mucosal morphological damage.

### 2.7 Serum cytokine levels analysis

Blood samples were collected immediately following mice sacrificed, underwent a 10-minute centrifugation at 3,000 rpm at 4°C for serum extraction, and supernatants were maintained at -80°C till further analyses. Murine ELISA kits (88-7064, Thermo Fisher, Austria; EK280/3-01, MuLTI SCIENCE, Shanghai) were employed for D-lactic acid, Interleukin (IL)-1β, and IL-6 detection, following kit protocols.

### 2.8 Immunofluorescent assessment of tight junction

Following fixation and permeabilization in methanol or acetone at 20°C, intestinal tissues were overnight (ON) exposed to primary antibodies at 4°C, then treated with FITC-labeled secondary antibody for 1 hour at RT. Following nuclear counterstaining, the slices were treated to mounting media with 4,6-diamidino-2-phenylindole (DAPI), prior to visualization and image capture under a fluorescence microscopy. DAPI and FITC images were captured from the same tissue section.

### 2.9 Quantitative PCR

Murine intestinal samples were collected, and flash-frozen in liquid nitrogen, before storage at -80°C till further analyses. Total RNA isolation was conducted using TRIzol (15596026, Invitrogen, Carlsbad, CA, USA), and quantification *via* the Universal SYBR FAST qPCR Kit Master Mix (2x) (KAPA Biosystems, USA). The qPCR reaction parameters were as follows: 10 minutes at 95 °C, 45 cycles for 10 seconds at 95 °C, and 60 seconds at 59 °C, then 15 seconds at 95 °C, 15 seconds at 72 °C, and 15 seconds at 95 °C. Relative gene expression of Zonula occludens-1 (*ZO-1*), *Occludin*, and *Gapdh* were assessed *via* the 2^-ΔΔCt^ formula. The employed primer sequences are as follows: *ZO-1* forward 5′-GAGCAAGCCTCC-5′-GAGCAAGCCTCC-5′-GAGCAAGCCTCC-5′-GAGCAATGCACATA-3′, reverse 5′-TCAGTTTCGGGTTTCCTT-3′; *Occludin* forward 5′-CAACGGCAAAGTGAATGGCA-3′, reverse 5′-CTTTCCTTCGTGGGAGTC-3′; *Gapdh* forward 5′-TGTGAACGGATTTGGCCGTA-3′, reverse 5′-GATGGTGATG GGTTTCCCGT-3′.

### 2.10 Western blot analysis

Murine intestines underwent lysis in lysis buffer, and protein quantification was performed *via* a BCA kit (Beyotime, China). Equal protein amounts were then electrophoresed on SDS/PAGE in a Bio-Rad Mini-PROTEAN apparatus, prior to transfer to PVDF membranes (Bio-Rad, Marnes-la-Coquette, France), which then underwent a 1-hour blocking in 5% nonfat milk (w/v) at RT, with subsequent ON exposure to primary antibodies at 4°C. The employed primary antibodies are listed as follows: anti-Occludin antibody (13409-1-AP); anti-ZO-1 antibody (21773-1-AP); and anti-GAPDH antibody (60004-1-Ig). All aforementioned antibodies were used in a 1:1000 dilution, and were purchased from Proteintech, USA. Subsequently, the separated proteins were treated with secondary antibodies: HRP-goat anti-mouse IgG (115-035-003) and HRP-goat anti-rabbit IgG (111-035-144) from Jackson ImmunoResearch. Protein band visualization was done with ECL chemiluminescence imaging system, and quantification *via* ImageJ software (Version 1.50i; National Institutes of Health, Bethesda, MD, USA). Finally, we calculated the IntDen (target protein)/IntDen (GAPDH) ratios.

### 2.11 Lactate dehydrogenase cytotoxicity assay

Target cell cytotoxicity was assessed based on the cellular LDH release, using an LDH cytotoxicity detection kit, following kit directions (TaKaRa, Japan). The LDH release percentage was computed as follows: % release =100×(experimental LDH release–spontaneous LDH release)/(maximal LDH release–spontaneous LDH release). 1% Triton X-100-treated cells were employed as positive controls for maximal LDH release.

### 2.14 Statistical analysis

Data are provided as mean maximal LDH 100ed on thees of Health, Bethesda, Msessed with the one-way analysis of variance (ANOVA), and inter-group comparisons were assessed using the t-test. After feature selection and data preprocessing, we developed 5 popular ML-based models to predict sepsis. Overall performance of each model was assessed *via* the accuracy, precision, and F1-measure. The best performing model was applied to the further interpretation. Finally, SHAP summary analysis, SHAP dependence analysis was utilized for model explainability. Statistical analyses were conducted using SPSS statistical software version 24.0 (IBM Corp., Armonk, NY, USA), R statistical software version 3.6.1 (R Project for Statistical Computing, Vienna, Austria), and Python software version 3.6.6 (Python Software Foundation, Wilmington, DE, USA). All statistical tests were two-sided, and *P*-values less than 0.05 were considered to be statistically significant.

## 3 Results

### 3.1 Alterations in serum metabolome among septic patients

**Figure 1:**
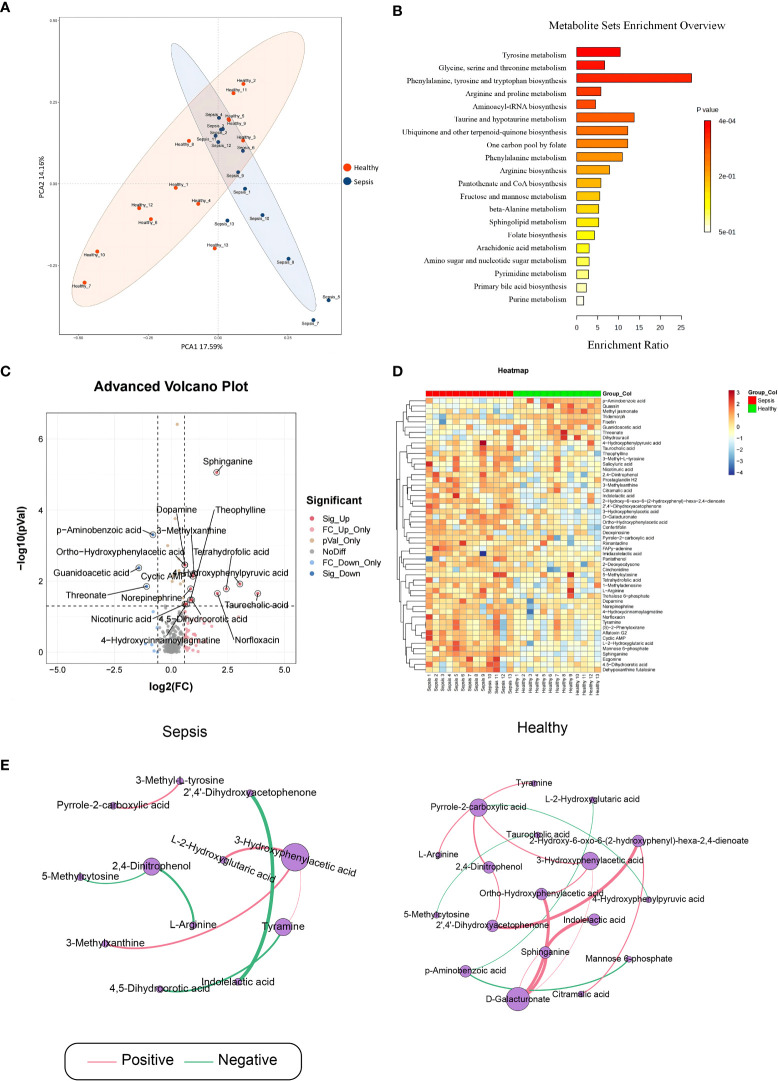
Analysis of sepsis-related serum metabolites. Serum samples were obtained from septic patients and healthy individuals. **(A)** Principal Component Analysis **(B)** The metabolite sets enrichment analysis **(C)** Volcano plot for differentially regulated metabolites between control and sepsis groups. **(D)** Heat map of differentially regulated metabolites. **(E)** significant pearson correlation.

To detect alterations in serum metabolome during early sepsis, we conducted LC-MS analysis, which identified 507 metabolites among 26 analyzed serum samples. The PCA scatter plots (**Figure 1A**) demonstrated that the metabolomics analysis was of high quality, with clustered QC samples. Based on our KEGG network enrichment analysis of differentially regulated metabolites between the sepsis and healthy cohorts (Fisher exact test), there were marked alterations in multiple signal transduction networks, like those involving tryptophan, glycine, serine and threonine, and pyrimidine metabolisms (**Figure 1B**). Using volcano plot filtering, we next revealed marked differentially regulated metabolites between the two cohorts (**Figure 1C**). A heatmap of the metabolites illustrated that the differentially regulated metabolites were heavily clustered in each cohort (**Figure 1D**). The pearson correlation analysis method was employed to examine variations in the metabolite data between the two different groups, with the aim of identifying any significant associations or correlations (**Figure 1E**).

### 3.2 Model performance

**Figure 2:**
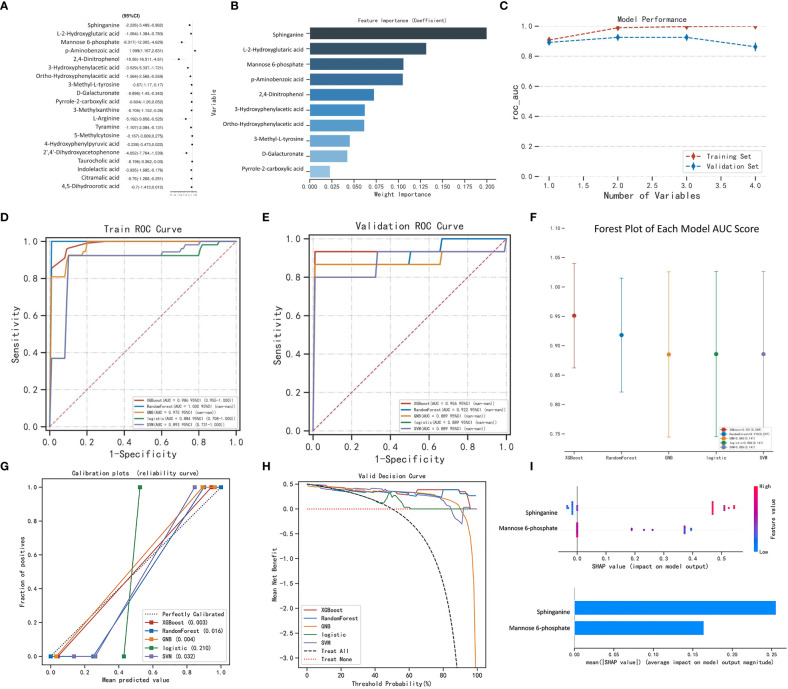
Machine learning model performance. Linear regression analysis. **(B)** XGBoost model: feature importance. **(C)** Model performance. Receiver-operating characteristic curves for 5 machine learning models. The XGBoost model achieved a larger (better) AUROC compared with the other models: **(D)** Train ROC curve, **(E)** Validation ROC curve. **(F)** Forest plot of the AUC Score of the 5 models. **(G)** Calibration plots of 5 models. The XGBoost achieved lower (better) Brier scores compared with the other models. **(H)** Decision curve analysis for machine learning models. **(I)** SHAP analysis was performed on the XGBoost model to visually represent the importance of each feature. Each feature is represented by a color that corresponds to the variable’s value, with red indicating a larger value and blue indicating a smaller value. This analysis provides insight into the relationship between each feature and its importance in the model. **(A)**.

The variables that showed statistically significant differences in the single-factor analysis were subjected to multi-factor analysis using linear regression (**Figure 2A**). To score the variable sets, the XGBOOST algorithm was utilized. The scoring process involved adding variables sequentially, starting with Sphinganine, L-2-Hydroxyglutaric acid, Mannose 6-phosphate, p-Aminobenzoic acid, 2,4-Dinitrophenol, 3-Hydroxyphenylacetic acid, Ortho-Hydroxyphenylacetic acid, 3-Methyl-L-tyrosine, D-Galacturonate, and Pyrrole-2-carboxylic acid. The order of variables in each set was determined by their importance, which was estimated prior to scoring. The best set of variables identified through this process was Sphinganine and Mannose 6-phosphate (**Figures 2B, C**). The XGBoost model outperformed the other models with a higher AUROC compared with other 4 models, indicating better performance (**Figures 2D, E**; **Tables 1**, **2**). Based on the AUROC of the 5 models, we made a forest plot of the AUC score of the multiple models. 5 models were seen after using 5 cross- validations, results showed that the XGBoost model e has the most stable performanc (**Figure 2F**). Based on the above aspects, we can conclude that the XGBoost model(AUROC=0.956) significantly outperformed 4 other machine learning models. The calibration plots of the five models are shown in **Figure 2G**. DCA indicated that the XGBoost model could serve as the best diagnostic tool for sepsis in **Figure 2H**. The SHAP package was utilized to analyze the XGBoost model, which demonstrated the impact of each feature on the sample and identified both positive and negative influences. The resulting bar chart displayed the correlation between the feature value’s magnitude and its predicted impact (**Figure 2I**).

**Table 1:** Performance metrics for five models in the training dataset.

Model	AUC (SD)	Accuracy (SD)	Sensitivity (SD)	Specificity (SD)	PPV (SD)	NPV (SD)	F1 score (SD)	Kappa (SD)
XGBoost	0.986 (0.006)	0.885 (0.023)	0.907 (0.081)	0.964 (0.045)	0.960 (0.049)	0.838 (0.063)	0.928 (0.023)	0.771 (0.044)
RandomForest	1.000 (0.000)	0.942 (0.019)	1.000 (0.000)	1.000 (0.000)	1.000 (0.000)	0.897 (0.032)	1.000 (0.000)	0.885 (0.037)
GNB	0.975 (0.019)	0.885 (0.037)	0.964 (0.073)	0.905 (0.086)	0.910 (0.078)	0.875 (0.054)	0.931 (0.039)	0.771 (0.074)
logistic	0.884 (0.037)	0.875 (0.022)	0.924 (0.038)	0.924 (0.038)	0.918 (0.041)	0.842 (0.034)	0.920 (0.023)	0.750 (0.044)
SVM	0.893 (0.037)	0.875 (0.022)	0.924 (0.038)	0.924 (0.038)	0.918 (0.041)	0.842 (0.034)	0.920 (0.023)	0.750 (0.044)

PPV, Positive Predictive Value; NPV, Negative predictive value; XGBoost, eXtreme Gradient Boosting; SVM, support vector machines; SD, Standard Deviation.

**Table 2:** Performance metrics for five models in the validation dataset.

Model	AUC (SD)	Accuracy (SD)	Sensitivity (SD)	Specificity (SD)	PPV (SD)	NPV (SD)	F1 score (SD)	Kappa (SD)
XGBoost	0.956 (0.089)	0.887 (0.157)	0.933 (0.133)	1.000 (0.000)	1.000 (0.000)	0.850 (0.200)	0.960 (0.080)	0.790 (0.283)
RandomForest	0.922 (0.097)	0.887 (0.157)	0.867 (0.163)	1.000 (0.000)	1.000 (0.000)	0.850 (0.200)	0.920 (0.098)	0.790 (0.283)
GNB:Gaussian Naive Bayes;	0.889 (0.141)	0.860 (0.196)	0.867 (0.163)	1.000 (0.000)	0.900 (0.200)	0.833 (0.211)	0.874 (0.170)	0.723 (0.391)
logistic	0.889 (0.141)	0.893 (0.137)	0.933 (0.133)	0.933 (0.133)	0.933 (0.133)	0.867 (0.163)	0.920 (0.098)	0.790 (0.273)
SVM	0.889 (0.141)	0.893 (0.137)	0.933 (0.133)	0.933 (0.133)	0.933 (0.133)	0.867 (0.163)	0.920 (0.098)	0.790 (0.273)

PPV, Positive Predictive Value; NPV, Negative predictive value; XGBoost, eXtreme Gradient Boosting; SVM, support vector machines; SD, Standard Deviation.

### 3.3 Analysis of the correlation between the expression of metabolites and the severity of sepsis

**Figure 3:**
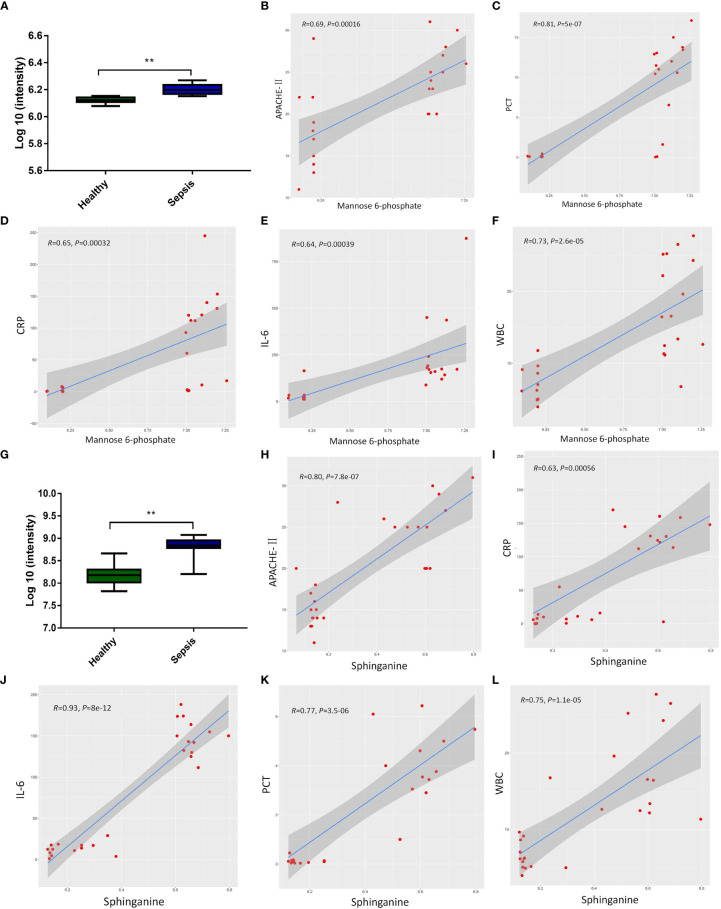
The correlation between the expression of metabolites and the severity of sepsis. **(A)** Relative expression of sphinganine in healthy group and sepsis group. **(B-F)** Pearson correlation of the expression of sphinganine and APACHE-II(R=0.69, *P*<0.001), PCT(R=0.81, P<0.001), CRP(R=0.65, P<0.001), IL-6(R=0.64, P<0.001), WBC(R=0.73, P<0.001). **(G)** Relative expression of Mannose 6-phosphate in healthy group and sepsis group. **(H-L)** Pearson correlation of the expression of mannose-6-phosphate and and APACHE-II(*R*=0.80, *P*<0.001), CRP(*R*=0.63, *P*<0.001), IL-6(*R*=0.93, *P*<0.001), PCT(*R*=0.77, *P*<0.001), WBC(*R*=0.75, *P*<0.001). *P* values 0.05 (*) or *P* values 0.01 (**) was regarded as significant.

To investigate the relationship between metabolites (Sphinganine and Mannose 6-phosphate) and the severity of sepsis, we analyzed the relative expression levels of metabolites in the healthy group and sepsis group, as well as the correlation between metabolites and Acute Physiology and Chronic Health Evaluation-II(APACHE-II) score, PCT (μg/L), white blood cell (WBC)×10^9^/L, CRP(mg/L), and Interleukin-6(IL-6) (pg/ml). Relative expression of sphinganine in healthy group and sepsis group (**Figure 3A**). Furthermore, the expression of sphinganine and APACHE-II(*R*=0.69, *P*<0.001), PCT(*R*=0.81, *P*<0.001), CRP(*R*=0.65, *P*<0.001), IL-6(*R*=0.64, *P*<0.001), WBC(*R*=0.73, *P*<0.001), showed a strong positive correlation (**Figures 3B–F**). Relative expression of Mannose 6-phosphate in healthy group and sepsis group (**Figure 3G**). Furthermore, the expression of sphinganine and APACHE-II(*R*=0.80, *P*<0.001), CRP(*R*=0.63, *P*<0.001), IL-6(*R*=0.93, *P*<0.001), PCT(*R*=0.77, *P*<0.001), WBC(*R*=0.75, *P*<0.001), showed a strong positive correlation(**Figures 3H–L**).

### 3.4 Serum metabolite sphinganine alleviates LPS-induced intestinal epithelial cell injury *in vitro*

**Figure 4:**
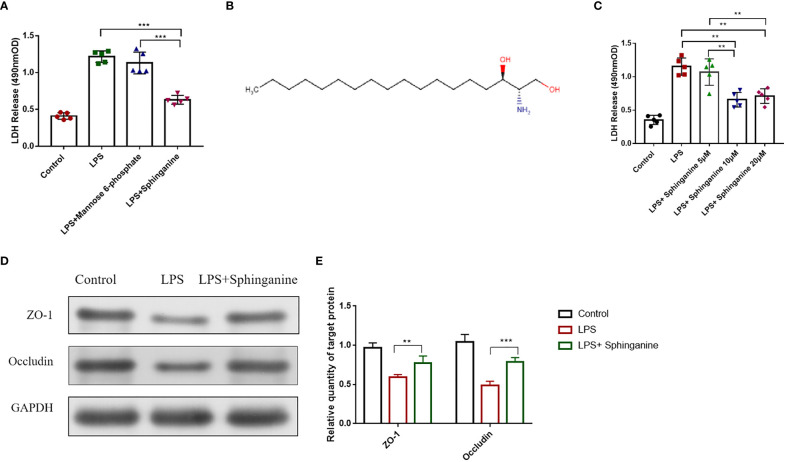
Sphinganine alleviates sepsis-induced intestinal injury *in vitro.* **(A)** Effect of a 24 h treatment with mannose-6-phosphate (10μm) and sphinganine (10μm) on Caco-2 cell cytotoxicity, as evidenced by the LDH assay. **(B)** Chemical structure of sphinganine. **(C)** Outcome of 24 h treatments with varying sphinganine concentrations on Caco-2 cell cytotoxicity, as evidenced by the LDH assay. **(D)** ZO-1 and occludin protein expressions, as assessed by Western blot analysis. **(E)** Statistical plot of gray value of ZO-1 and Occludin were detected by Western blot. *P* values 0.05 (*) or 0.01 (**) was regarded as significant.

We employed the Caco-2 monolayer cell culture model to validate the mannose-6-phosphate- and sphinganine-mediated protection of the intestinal epithelium *in vitro.* Upon sphinganine treatment, LDH levels were significantly reduced (**Figure 4A**). Based on the metabolome database (HMDB), sphinganine (HMDB00296) is a phosphatidic acid molecule (C_18_H_39_NO_2_) with a molecular weight of 301.5078Da (**Figure 4B**). To establish the optimal treatment concentration of sphinganine in Caco-2 cells, we performed the LDH release assay. Based on our results, the optimal dosage was 10μM sphinganine over a 48h period (**Figure 4C**). We also observed that the Occludin and ZO-1 contents were strongly enhanced in the LPS + sphinganine cohort, compared to the LPS cohort, thereby confirming the sphinganine-mediated protection of the colonic mucosal barrier from LPS-induced damage (**Figures 4D, E**).

### 3.5 Serum metabolite sphinganine alleviates sepsis-induced intestinal injury *in vivo*

**Figure 5:**
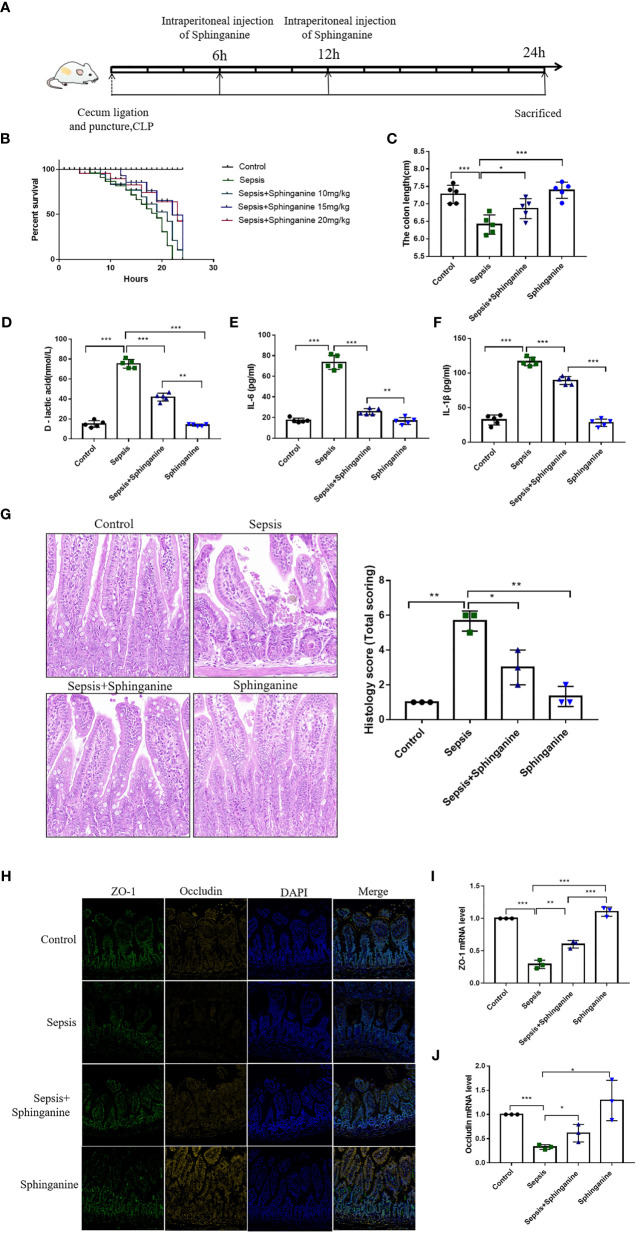
Sphinganine alleviates sepsis-induced intestinal injury *in vivo.* **(A)** Design of animal experiment. **(B)** Effect of varying sphinganine concentrations on the SR of sepsis mice. **(C)** Colon length. Serum levels of **(D)** D-lactic acid, **(E)** IL-6, and **(F)** IL-1β. **(G)** Colon tissues stained with HE and histopathological scores analysis from slides. **(H)** Immunofluorescent staining of intestinal TJ proteins, namely, ZO-1 and occludin (scale bar, 50um). **(I, J)** Intestinal ZO-1 and occludin gene expression analysis *via* qPCR. Mann-Whitney U test was employed for comparison. *P* values 0.05 (*), 0.01 (**) or 0.001 (***) was regarded as significant.

To further explore the sphinganine-mediated protection of sepsis-induced intestinal injury, we established a sepsis animal model (**Figure 5A**). Based on our 0-24h observations, control mice exhibited a 100% survival rate (SR), whereas, sepsis mice exhibited a 15% SR at 24 h. Among the sepsis + sphinganine (10 mg/kg) mice, the SR was 22% at 24 h, whereas, in mice receiving increasing amounts of sphinganine (15 and 20 mg/kg) with sepsis, the SRs were 65.42% and 63.48%, respectively, at 24 h. Given these evidences, sphinganine at 15 mg/kg strongly diminished septic mice mortality in a dose-dependent manner (**Figure 5B**). In sepsis mouse, shortened colon length is a strong biomarker of colon inflammation severity. Relative to the sepsis mice, mice treated with sphinganine exhibited strongly enhanced colon length (*p* < 0.05) (**Figure 5C**), as well as markedly diminished D-lactic acid, IL-1β, and IL-6 contents (*p* < 0.05) (**Figures 5D–F**). Herein, we employed the Chiu pathological mucosal injury score to measure the extent of intestinal histological damage. In the control and sphinganine-treated mice, we observed normal epithelial cells without ulcers, a large percentage of goblet cells, a close arrangement of large intestinal glands, and a normal morphology of the colonic mucosa. In contrast, the sepsis mice exhibited ulcers in the colonic mucosa superficial layer, with complete disappearance of the mucosal tissue layer, and strong infiltration by inflammatory cells. Alternately, the sepsis + sphinganine mice showed no inflammatory cell infiltration, ulcers, or epithelial cell damage, and the quantity of goblet cells was vastly diminished. Based on the Chiu pathological scoring system, the sepsis + sphinganine mice had considerably less intestinal mucosa damage, compared to the sepsis mice (**Figures 5G**). In addition, the ZO-1 and Occludin protein expressions were enhanced among sepsis + sphinganine mice, relative to the sepsis mice. This indicates that sphinganine, indeed, protects the colon mucosa barrier from sepsis-induced damage. We further confirmed our findings using immunofluorescence immunostaining and qPCR (**Figures 5H–J**).

## 3 Discussion

Herein, serum samples were collected from healthy individuals and septic patients for metabolome analysis. Based on our results, the relative gene expression of p-Aminobenzoic acid, Methyl jasmonate, Tridemorph, Fisetin, Guanidoacetic acid, Threonate, Dihydrouracil, and 4-Hydroxyphenylpyruvic acid were markedly enhanced among healthy individuals, relative to septic patients, thereby suggesting that the early stage of sepsis likely involves metabolite dysregulation. The XGBOOST algorithm was utilized to screen the variable sets. The results revealed the metabolites mannose-6-phosphate and sphinganine as the optimal sepsis-related variables. A diagnostic model was established by five machine learning methods, namely XGBoost, RandomForest, GNB, logistic, and SVM, finally we found that the XGBoost model has the most stable performance. Pearson analysis reinforced the expression of Sphinganine, Mannose 6-phosphate were positively associated with the APACHE-II, PCT, WBC, CRP, and IL-6. Moreover, we explored the physiological roles the aforementioned metabolites. We demonstrated that sphinganine strongly diminished the LDH content in LPS-treated Caco-2 cells. In addition, using both *in vitro* and *in vivo* examination, we revealed that sphinganine strongly protects against sepsis-induced intestinal barrier injury.

Sepsis is a systemic inflammatory response that leads to systemic inflammation and multi-organ failure (14, 15). Despite years of research and clinical trials, there is still no reliable therapy targeting the dysregulated and inflammatory response that characterizes sepsis. The current manual assessment of sepsis using screening tools, such as the (Sequential Organ Failure Assessment)SOFA score for ICU patients, can be complicated due to the number of clinical signs measured, and may also lack sufficient sensitivity (16). On contrast, automated decision support systems based on artificial intelligence (AI) and machine learning, which utilize electronic health record (EHR) data, have shown a marked improvement in adherence to treatment protocols in ICUs (17). Herein, we employed the XGBOOST algorithm to screen the variable sets and the XGBoost model has the most stable performance in constructing the machine learning methods of sepsis. Machine learning (ML) models are often considered to be a “black box” in which data goes in and decisions come out, but the processes that occur between input and output are not transparent. In this study, we employed the SHAP value to interpret our XGBoost model, the result revealed that Sphinganine and Mannose 6-phosphate were the primary factors that contributed to the XGBoost model. Previous study has reported the ML models showed a good prognostic prediction ability in septic patients requiring ICU readmission (18). Another study reported that the study aimed to develop a high-performance machine learning sepsis prediction algorithm based on routinely collected intensive care unit data, designed to be implemented in European intensive care units, the result showed that the algorithm uses 4 hours of input and can identify patients with high risk of developing sepsis, with high performance (area under the receiver operating characteristics curve 0.90; area under the precision-recall curve 0.62) for predictions up to 3 hours before sepsis onset (19).

Following sepsis, there is a rise in intestinal permeability, which can lead to the translocation of intestinal bacteria and endotoxins. This process can exacerbate the sepsis and worsen the overall condition of the individual (20, 21). Hence, it is crucial to develop effective measures of sepsis-induced intestinal barrier injury prevention and treatment (22, 23). Currently, the treatment of intestinal injury involves several approaches such as protopathy, anti-infective therapy, immune regulation, and organ support and protection. However, despite these efforts, the effectiveness of the treatment remains limited and the mortality rate remains high (24). Ultimately, finding new strategies to treat intestinal injury will be critical for improving outcomes for patients with sepsis.

With advancements in metabolomics, there is a substantial increase in functional metabolites exploration and discovery (25, 26). For instance, bile acids, which are critical for immune regulation, were also shown to regulate the balance between TH17 and Treg cells using receptors like the farnesoid x (FXR) and G-protein coupled bile acid receptors (TGR5) (27, 28). Similarly, 3-indolepropionic acid, a derivative of gut microbiota tryptophan metabolism, also serves as an anti-inflammatory agent which protects the intestinal barrier integrity (29). In this study, we demonstrated that sphinganine protected against sepsis-induced intestinal barrier injury. Prior investigations revealed that sphinganine is a synthetic bioactive sphingolipid that inhibits C. glabrata and C. albicans development (30). This is the first report to demonstrate a protective role of sphinganine in the intestines. However, further research is needed to fully understand the mechanism by which sphinganine protects against intestinal damage.

In conclusion, our analysis of serum metabolites revealed that sepsis causes a strong dysregulation in serum metabolites. Based on our ML findings, serum metabolites not only have a good value in sepsis diagnosis, but also possess a protective value against sepsis-induced intestinal barrier injury. Our findings highlightedthe potential diagnostic value of the ML, and also provided new insight into enhanced therapy and/or preventative measures against sepsis. However, the number of patients in the sample is relatively small, and large sampling sizes are needed to comprehensively assess the diagnostic value of metabolites for sepsis.

## Data availability statement

The datasets presented in this study can be found in online repositories. The names of the repository/repositories and accession number(s) can be found below: MTBLS7878 (Metabolights).

## Ethics statement

The studies involving human participants were reviewed and approved by the Shanghai Fifth People’s Hospital. The patients/participants provided their written informed consent to participate in this study. The animal study was reviewed and approved by the Animal Center of East China Normal University of Shanghai.

## Author contributions

TJ conceived and designed the project. ZW wrote the manuscript. YQ provided data analysis support. FW contributed to clinical sample collection. BZ supervised the study. All authors contributed to the article and approved the submitted version.
